# Supplementary material for: Metalloprotease-Dependent S2′-Activation Promotes Cell–Cell Fusion and Syncytiation of SARS-CoV-2
Source: Viruses. 2022 Sep 21;14(10):2094. doi: 10.3390/v14102094 (PMC9608990; doi:10.3390/v14102094)
Supplement: Supplementary file 1 [file viruses-14-02094-s001.zip › viruses-1899253-supplementary.pdf]

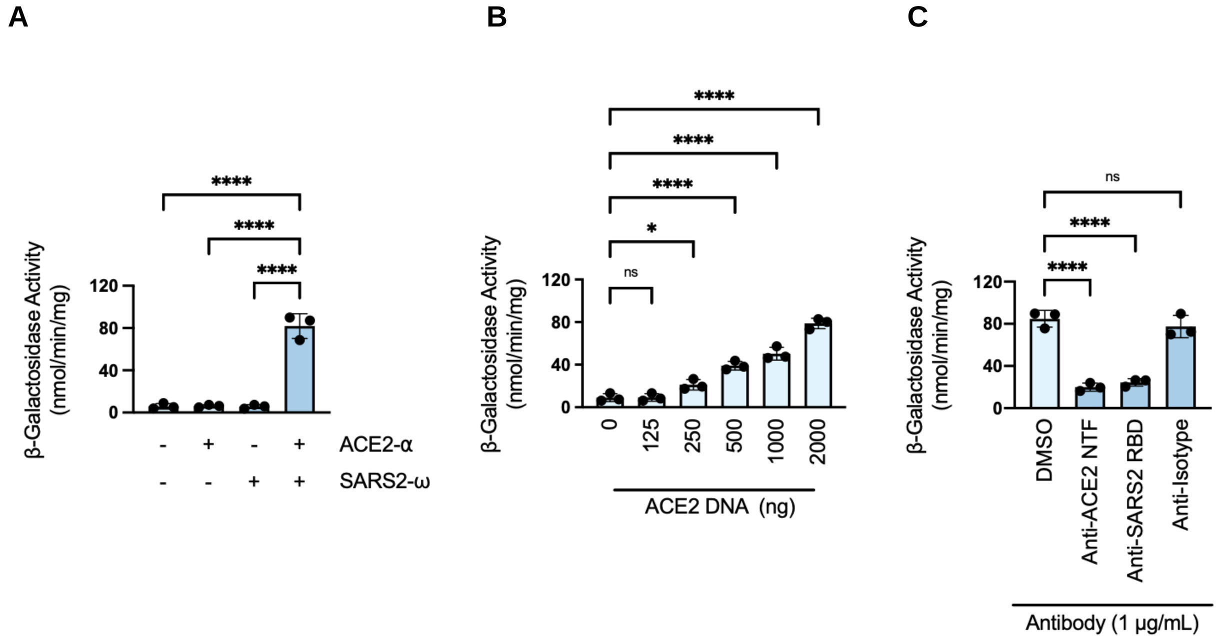

Figure S1. Validation of a 'split β-galactosidase' reporter for the quantitative *in vitro* measurement of SARS-CoV-2 spike protein-induced cell-cell fusion and syncytiation. **(A)** Target HEK293T cells were transiently co-transfected with human ACE2 and the α-fragment of β-galactosidase and co-culture with effector HEK293T cells transiently co-transfected with SARS-CoV-2 spike protein (SARS2) and the ω-fragment of β-galactosidase. Upon cell-cell fusion, active β-galactosidase is formed. Fusion-associated β-galactosidase activity at 16-hours post-co-cultured was measured in whole cell extracts by cleavage of a chromogenic substrate. **(B)** SARS-CoV-2 cell-cell fusion and syncytiation as measured by the 'split β-galactosidase' reporter is dependent on the presence of ACE2. Target HEK293T cells were transiently co-transfected with different amounts of human ACE2 expression plasmid (range: 0 ng - 2000 ng per well) and the α-fragment of β-galactosidase, and co-culture with effector HEK293T cells transiently co-transfected with SARS2 and the ω-fragment of β-galactosidase. The overall concentration of DNA per transfection was maintained equal with pcDNA3.1 empty vector. **(C)** SARS-CoV-2 cell-cell fusion and syncytiation as measured by the 'split β-galactosidase' reporter is dependent on the interaction of ACE2 and SARS2. Target HEK293T cells were transiently co-transfected with human ACE2 and the α-fragment of β-galactosidase and co-culture with effector HEK293T cells transiently co-transfected with SARS2) and the ω-fragment of β-galactosidase, in presence of either an anti-ACE2 N-terminal-specific (NTF) antibody, and anti-SARS-CoV-2 receptor bidding domain-specific (SARS2 RBD), or an anti-isotope antibody. Probability values: *, $p < 0.05$; ****, $p < 0.0001$.

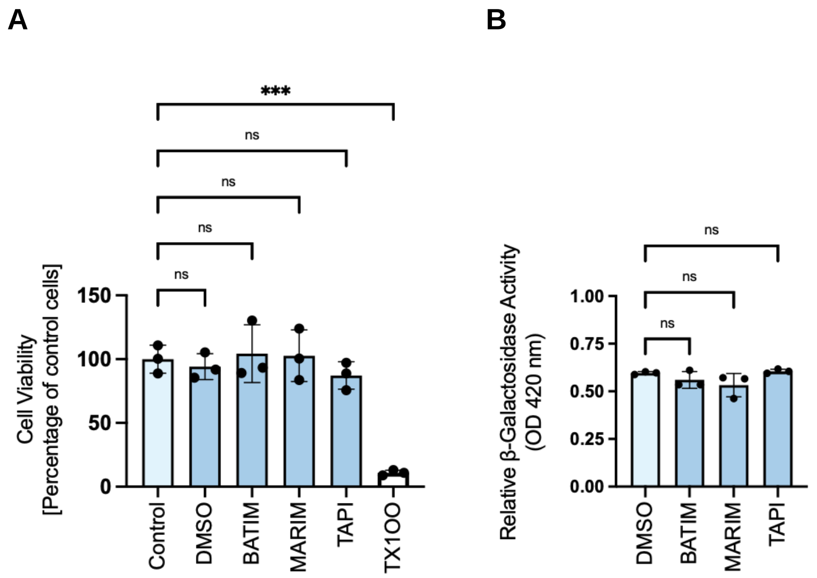

Figure S2. Hydroxamate-based metalloprotease inhibitors do not influence the 'split β-galactosidase' cell-cell fusion assay. **(A)** Target HEK293T cells were transiently co-transfected with human ACE2 and the α-fragment of β-galactosidase and co-culture with effector HEK293T cells transiently co-transfected with SARS-CoV-2 spike protein (SARS2) and the ω-fragment of β-galactosidase. Syncytial co-cultures described above were treated at co-culture with hydroxamate-based metalloprotease inhibitors (batimastat (20 μM; 16-hours), marimstat (20 μM; 16-hours), or TAPI-1 (20 μM; 16-hours)); corresponding cells were untreated or treated with DMSO vehicle as negative control cells, or 1% Triton-X-100 (TX100) as positive control cells. Cell viability was quantified by MTT assay and expressed as a percentage of untreated control cells. **(B)** Hydroxamate-based metalloprotease inhibitors (batimastat (20 μM), marimstat (20 μM), or TAPI-1 (20 μM) were spiked into whole cell extracts of syncytial co-cultures of target HEK293T cells transiently co-transfected with human ACE2 and the α-fragment of β-galactosidase and effector HEK293T cells transiently co-transfected with SARS-CoV-2 spike protein (SARS2) and the ω-fragment of β-galactosidase. The effect of metalloprotease inhibitors on the fusion-associated β-galactosidase activity was measured. Fusion-associated β-galactosidase activity is expressed as the corrected optical density at 420 nM. BATIM: batimastat; MARIM: Marimastat; TAPI: TAPI-1. Probability value: ***, $p < 0.001$.

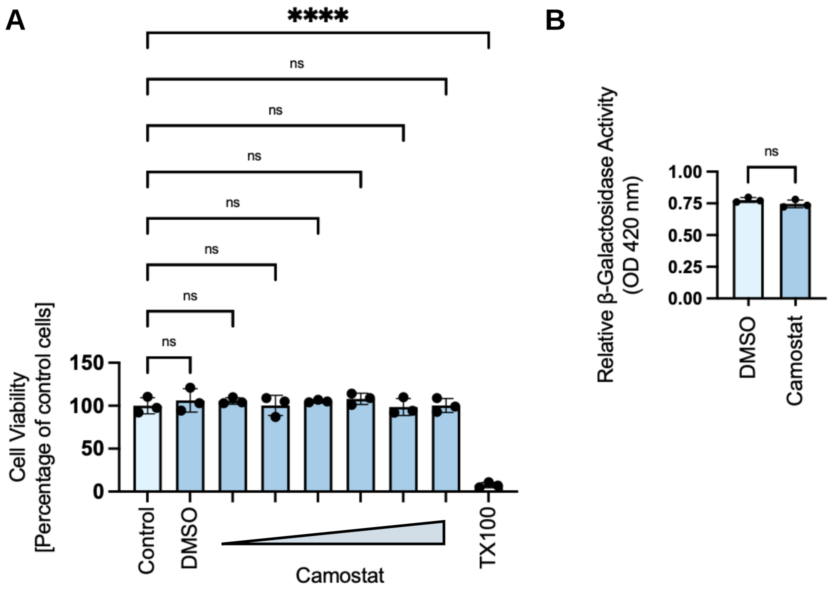

Figure S3. Camostat mesylate does not influence the 'split β-galactosidase' cell-cell fusion assay. **(A)** Target HEK293T cells were transiently co-transfected with human ACE2 and the α-fragment of β-galactosidase and co-culture with effector HEK293T cells transiently co-transfected with SARS-CoV-2 spike protein (SARS2) and the ω-fragment of β-galactosidase. Syncytial co-cultures described above were treated at co-culture with increasing concentrations of camostat mesylate (20 μM, 40 μM, 60 μM, 80 μM, 100 μM, 120 μM); corresponding cells were untreated or treated with DMSO vehicle as negative control cells, or 1% Triton-X-100 (TX100) as positive control cells. Cell viability was quantified by MTT assay and expressed as a percentage of untreated control cells. **(B)** Camostat mesylate (120 μM) was spiked into whole cell extracts of syncytial co-cultures of target HEK293T cells transiently co-transfected with human ACE2 and the α-fragment of β-galactosidase and effector HEK293T cells transiently co-transfected with SARS-CoV-2 spike protein (SARS2) and the ω-fragment of β-galactosidase. The effect of metalloprotease inhibitors on the fusion-associated β-galactosidase activity was measured. Fusion-associated β-galactosidase activity is expressed as the corrected optical density at 420 nM. CAMO: Camostat mesylate. Probability value: ****, $p < 0.0001$.

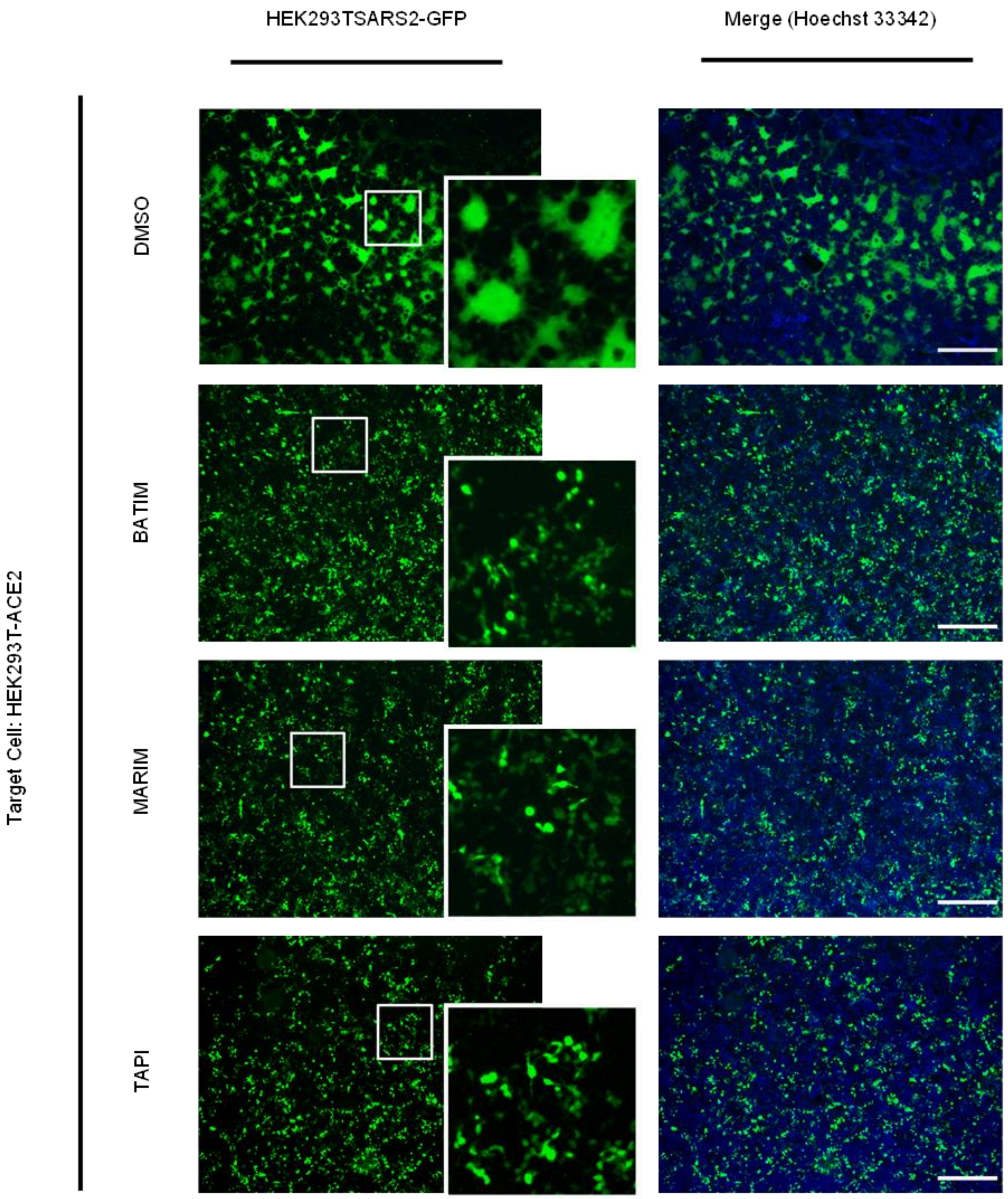

Figure S4. Expanded fluorescent micrographs from Figure 2 (Fig. 2D). Representative fluorescent micrographs of HEK293T-ACE2 and HEK293T-SARS2-GFP co-cultures post-treatment with hydroxamate-based metalloprotease inhibitors (BATIM: Batimastat (20 µM; 16-hours); MARIM: Marimastat (20 µM; 16-hours); TAPI: TAPI-1 (20 µM; 16-hours). Syncytiation was visualised at 16-hours post-co-culture on an EVOS FL Auto Imaging System (Thermo Fisher Scientific).

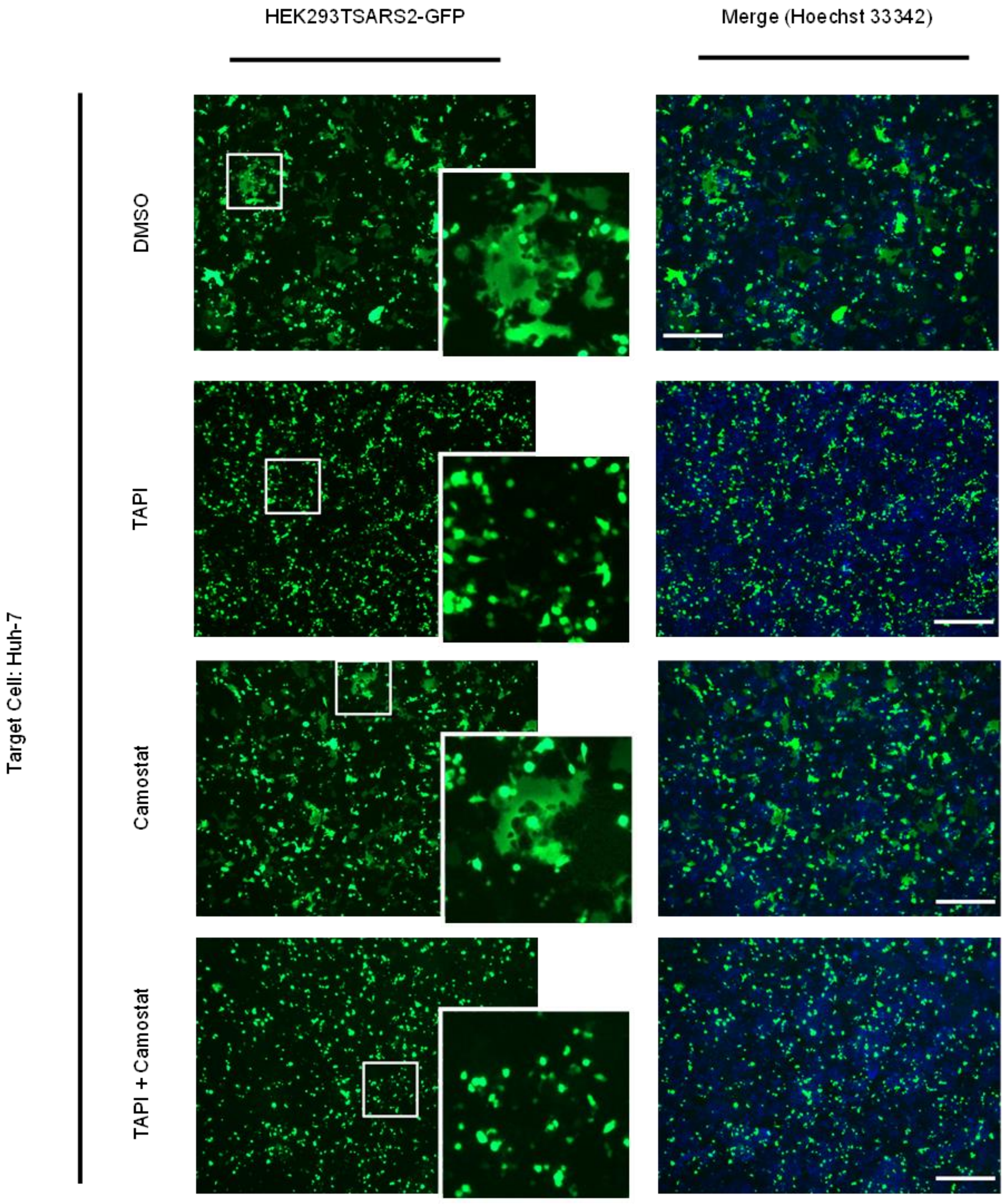

Figure S5. Expanded fluorescent micrographs from Figure 2 (Fig. 2F). Representative fluorescent micrographs of Huh-7 and HEK293T-SARS2-GFP co-cultures post-treatment with TAPI-1 (TAPI: 20 μM; 16-hours) and/or camostat mesylate (camostat: 120 μM; 16-hours). Syncytiation was visualised at 16-hours post-co-culture on an EVOS FL Auto Imaging System (Thermo Fisher Scientific).

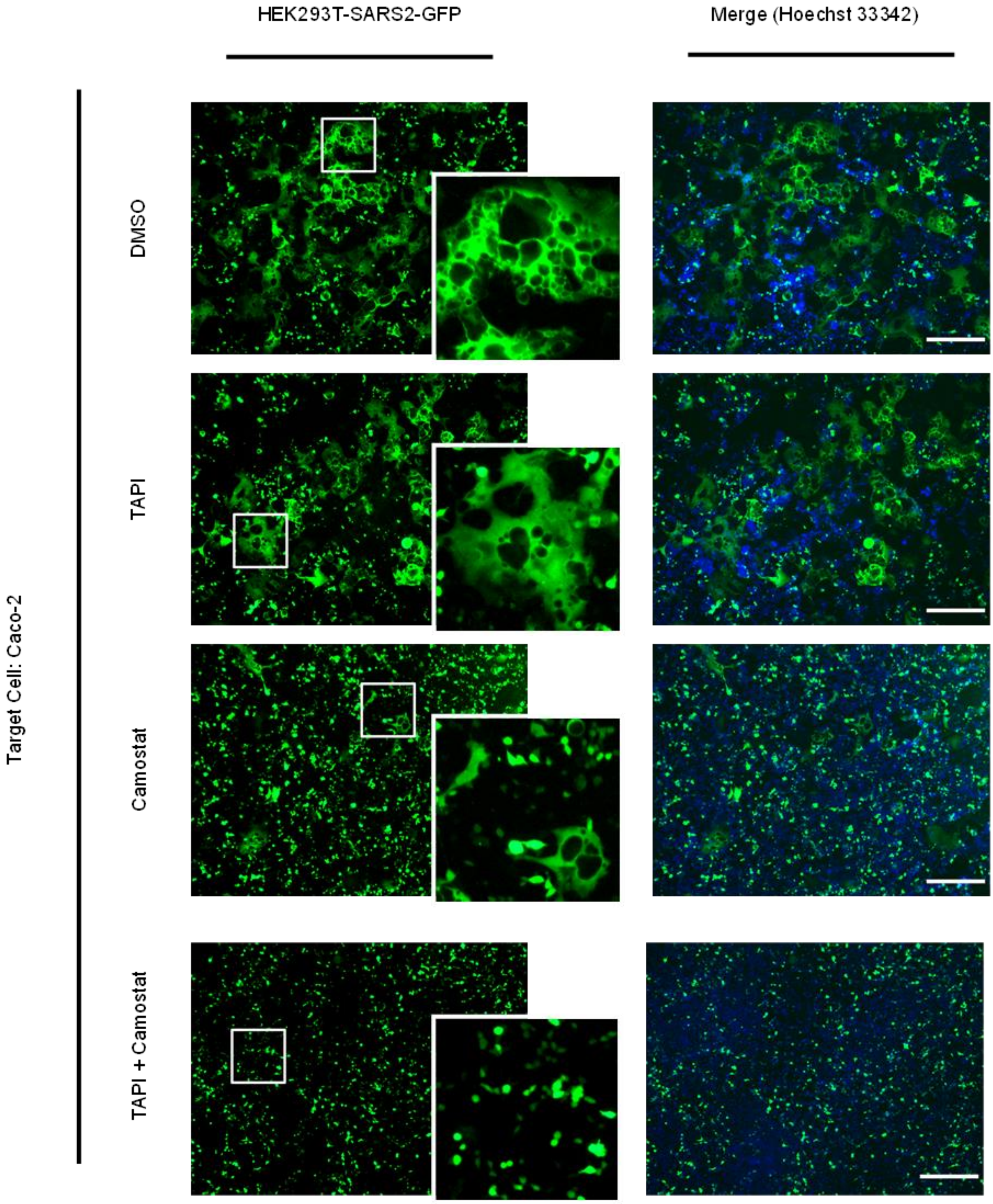

Figure S6. Expanded fluorescent micrographs from Figure 5 (Fig. 5E). Representative fluorescent micrographs of Caco-2 and HEK293T-SARS2-GFP co-cultures post-treatment with TAPI-1 (TAPI: 20 μM; 16-hours) and/or camostat mesylate (camostat: 120 μM; 16-hours). Syncytiation was visualised at 16-hours post-co-culture on an EVOS FL Auto Imaging System (Thermo Fisher Scientific).

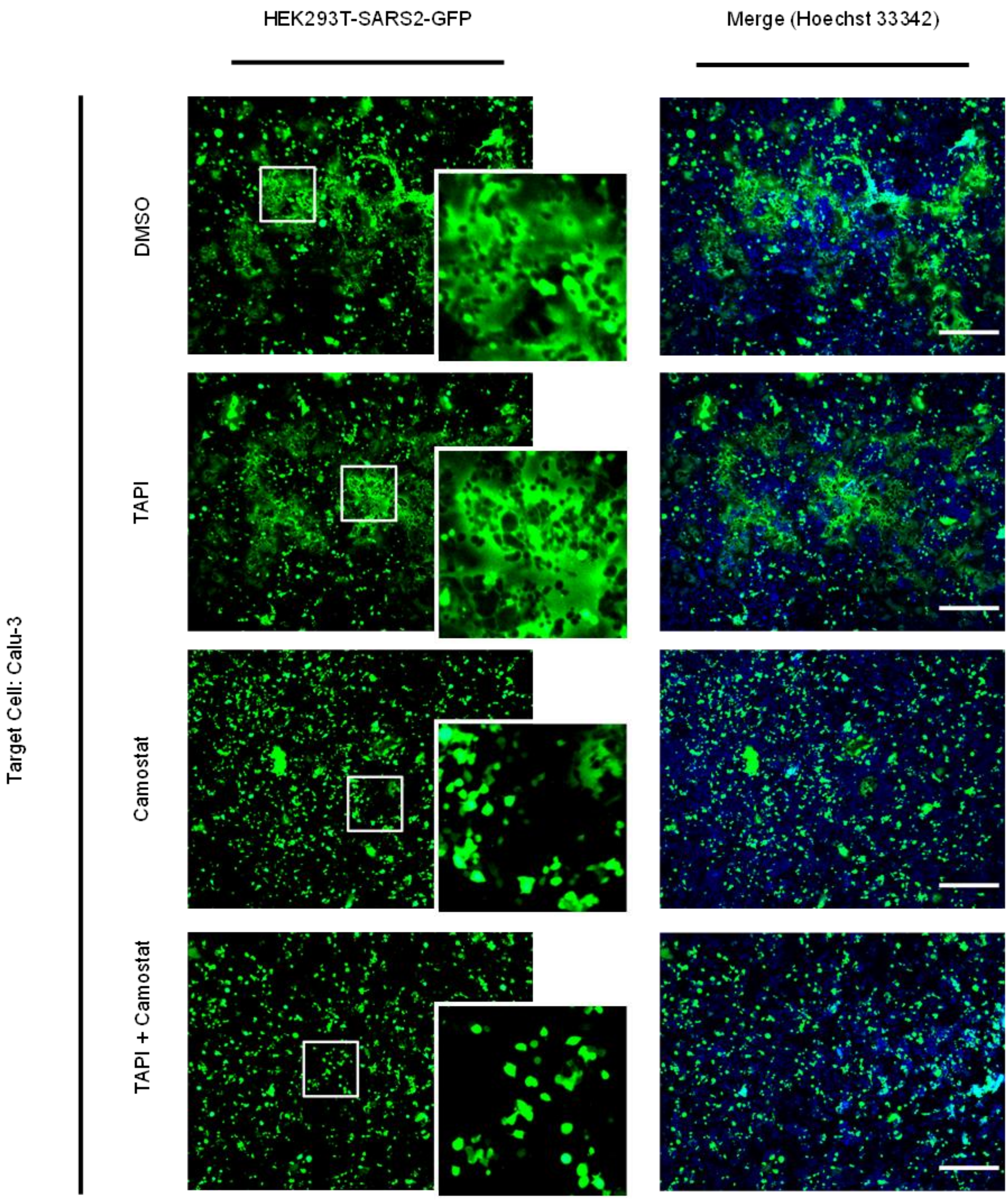

Figure S7. Expanded fluorescent micrographs from Figure 5 (Fig. 5G). Representative fluorescent micrographs of Calu-3 and HEK293T-SARS2-GFP co-cultures post-treatment with TAPI-1 (TAPI: 20 μM; 16-hours) and/or camostat mesylate (camostat: 120 μM; 16-hours). Syncytiation was visualised at 16-hours post-co-culture on an EVOS FL Auto Imaging System (Thermo Fisher Scientific).
